# Supplementary material for: Thalamic reticular control of local sleep in mouse sensory cortex
Source: eLife. 2018 Dec 25;7:e39111. doi: 10.7554/eLife.39111 (PMC6342525; doi:10.7554/eLife.39111)
Supplement: Transparent reporting form [file elife-39111-transrepform.docx]

***eLife’s* transparent reporting form**

We encourage authors to provide detailed information *within their submission* to facilitate the interpretation and replication of experiments. Authors can upload supporting documentation to indicate the use of appropriate reporting guidelines for health-related research (see [EQUATOR Network](http://www.equator-network.org/%20)), life science research (see the [BioSharing Information Resource](https://biosharing.org/)), or the [ARRIVE guidelines](http://www.plosbiology.org/article/info:doi/10.1371/journal.pbio.1000412) for reporting work involving animal research. Where applicable, authors should refer to any relevant reporting standards documents in this form.

If you have any questions, please consult our Journal Policies and/or contact us: [editorial@elifesciences.org](mailto:editorial@elifesciences.org).

**Sample-size estimation**

- You should state whether an appropriate sample size was computed when the study was being designed
- You should state the statistical method of sample size computation and any required assumptions
- If no explicit power analysis was used, you should describe how you decided what sample (replicate) size (number) to use

Please outline where this information can be found within the submission (e.g., sections or figure legends), or explain why this information doesn’t apply to your submission:

The number of animals/recordings per group was in agreement with the resource equation method to determine the sample size (Charan and Kantharia, 2013). No explicit power analysis was performed, as data analysis evolved in parallel with the acquisition of the data. Moreover sample size had to be adapted as a result of post-mortem histological analysis.

**Replicates**

1. You should report how often each experiment was performed
2. You should include a definition of biological versus technical replication
3. The data obtained should be provided and sufficient information should be provided to indicate the number of independent biological and/or technical replicates
4. If you encountered any outliers, you should describe how these were handled
5. Criteria for exclusion/inclusion of data should be clearly stated
6. High-throughput sequence data should be uploaded before submission, with a private link for reviewers provided (these are available from both GEO and ArrayExpress)

Please outline where this information can be found within the submission (e.g., sections or figure legends), or explain why this information doesn’t apply to your submission:

1. We report how often each experiment was performed in the figure legend of every panel of every figure that contains overviews and/or averages of the data that were collected. Additionally, a few datasets of particular relevance are repeated in the main text with specification of n number and mean +/- S.E.M. values. The few datasets not presented in the figures, such as paired-pulse ratios to identify TRN cells, are indicated with n number and mean data in the main text.
2. A biological replicate consisted of individual cells (for the *in vitro* part), or animals (for the *in vivo* part). A technical replicate consisted of the repetition of a current injection into a cell recorded at a given holding potential in patch clamp mode (*in vitro*), or a repeated treatment injection in the same animal (*in vivo*, chemogenetics). Technical replicates were meaned per biological replicate and are indicated in the text.
3. As mentioned, all biological replicate numbers are specified in the figure legends and/or in the main text. Technical replicate numbers are specified in the Methods.
4. For Figure 5 and 6, we excluded two outlier WT mice with S1 and S2 recording sites with an unusually small signal amplitude that prevented comparison to other mice (note that no normalization was applied for spindle detection).
5. Criteria for exclusion of cells (in vitro part) are a variation of the access resistance > 20% or a holding current at -60 mV < -150 pA. Criteria for exclusion of animals (in vivo part) was based on anatomical assessment of the localization of viral injection or recording site.
6. N/A

**Statistical reporting**

- Statistical analysis methods should be described and justified
- Raw data should be presented in figures whenever informative to do so (typically when N per group is less than 10)
- For each experiment, you should identify the statistical tests used, exact values of N, definitions of center, methods of multiple test correction, and dispersion and precision measures (e.g., mean, median, SD, SEM, confidence intervals; and, for the major substantive results, a measure of effect size (e.g., Pearson's r, Cohen's d)
- Report exact p-values wherever possible alongside the summary statistics and 95% confidence intervals. These should be reported for all key questions and not only when the p-value is less than 0.05.

Please outline where this information can be found within the submission (e.g., sections or figure legends), or explain why this information doesn’t apply to your submission:

We provide a full section in the Methods describing all of the above-mentioned points on statistical reporting. That is, all statistical analysis are described in the figure legends, with indication of sample size, type of statistical test used, and exact p-values. We also give p-values whenever these are non-significant. We also describe the rationale and motivation for using each of the statistical tests. Raw data are presented in figures whenever informative to do so and in particular when N per group is less than 10 (see e.g. Figure 4 and Figure 4 – supplement). Then, in the figures we indicate all p-values and sample size when appropriate. Our format to present data averages is mean +/- S.E.M. with individual datapoints typically superimposed. When data distributions only are presented, we chose either a Box-and-Whisker plot that includes the 95% percentile or, in one case, a cumulative plot for illustrative purposes to complement datasets otherwise analysed as means +/- S.E.M.

(For large datasets, or papers with a very large number of statistical tests, you may upload a single table file with tests, Ns, etc., with reference to sections in the manuscript.)

**Group allocation**

- Indicate how samples were allocated into experimental groups (in the case of clinical studies, please specify allocation to treatment method); if randomization was used, please also state if restricted randomization was applied
- Indicate if masking was used during group allocation, data collection and/or data analysis

Please outline where this information can be found within the submission (e.g., sections or figure legends), or explain why this information doesn’t apply to your submission:

Samples were randomly allocated to the groups. There was no restricted randomization. Experimenters were blinded to technical replicates of drug injections and analyses were carried out blind to the genotype of the animals.

**Additional data files (“source data”)**

- We encourage you to upload relevant additional data files, such as numerical data that are represented as a graph in a figure, or as a summary table
- Where provided, these should be in the most useful format, and they can be uploaded as “Source data” files linked to a main figure or table
- Include model definition files including the full list of parameters used
- Include code used for data analysis (e.g., R, MatLab)
- Avoid stating that data files are “available upon request”

Please indicate the figures or tables for which source data files have been provided:

Most of the relevant data files are presented in the figure showing the explicit raw data (Figure 3-6). Figure 2 contains in vitro cellular data presented in standard format. Additionally a “source data file” as excel document is provided for each figure and presents all numerical data, as well as corresponding detailed statistics. Titles and labels for each figure are also added in the excel “source data files”.
